# Occurrence and pattern of congenital heart diseases in a rural area of sub-Saharan Africa

**DOI:** 10.5830/CVJA-2010-046

**Published:** 2011-04

**Authors:** JC Tantchou Tchoumi, JC Ambassa, G Butera, A Giamberti, JC Sadeu

**Affiliations:** Cardiac Centre, St Elizabeth Catholic General Hospital, Shisong, Kumbo, Cameroon; Cardiac Centre, St Elizabeth Catholic General Hospital, Shisong, Kumbo, Cameroon; Paediatric Cardiac Surgery Department, Policlinico San Donato, Milan, Italy; Paediatric Cardiac Surgery Department, Policlinico San Donato, Milan, Italy; Department of Obstetrics and Gynaecology, Reproductive Biology Divison, McMaster University, Hamilton, Ontario, Canada

**Keywords:** congenital heart diseases, sub-Saharan Africa, ventricular septal defect, Cameroon

## Abstract

**Summary:**

The extent of congenital heart disease in Cameroon remains largely unknown. The aim of this study was to determine the occurrence and pattern of congenital heart diseases in the Cardiac Centre of St Elizabeth Catholic General Hospital, situated in a rural area of Cameroon.

**Methods:**

Between November 2002 and November 2008, a population of 2 123 patients with suspected cardiac pathologies were consulted at St Elizabeth Catholic General Hospital referral cardiac centre. Of these patients, 292 subjects were recruited for the study, based on detection of (1) precordial murmurs and/or cardiomegaly on chest X-ray examination, or (2) congenital heart diseases on transthoracic Doppler echocardiography examination.

**Results:**

Congenital heart diseases and inorganic murmurs were found in 95.5 and 4.5% of the patients, respectively. Congenital heart diseases included tetralogy of Fallot (26.1%), isolated ventricular septal defect (38.8%), atrioventricular cushion defect (7.3%), isolated atrial septal defect (2.8%), arterial duct cases (12.4%), common arterial trunk (1.3%), isolated stenosis of the pulmonary artery (2.6%), coarctation of the aorta (1.1%), congenital mitral valve regurgitation (1.2%), atresia of the triscupid valve (1.6%), double-outlet right ventricle (2.1%), anomalous pulmonary venous return (1.5%) and left isomerism (1.2%).

**Conclusion:**

Our data show that there is a high occurrence of congenital heart disease in this hospital in a rural zone of sub-Saharan Africa and that isolated ventricular septal defect is the predominant pathology. Post-surgical follow up remains very challenging as many parents cannot afford their children’s medical treatment or are generally not well educated.

## Summary

Congenital heart disease is defined as an abnormality in the cardio-circulatory structure or function, which is either present at birth or appears much later in life. The prevalence and pattern of this group of disorders varies both within and between regions and countries.^1^,^2^ However, the extent of its occurrence in Cameroon is largely unknown.^3^

In the majority of developing nations, and especially in most countries in the African continent, only a small and insignificant portion of the population can afford the cost of diagnosis, medical treatment and/or surgical correction of congenital heart diseases. The situation is even worse for those living in rural areas where access to basic healthcare is already a serious issue. Despite their wealth in natural resources, rural areas in developing countries are usually the poorest regions in terms of financial resources. These regions depend entirely on the availability of public health funding to finance and support their healthcare. Most of the time these funds do not reach them or are simply not provided.

The Shisong referral cardiac centre is specialised in both adult and paediatric cardiology and is part of Shisong’s St Elizabeth Catholic General Hospital, located in a rural area of the northwest province of Cameroon. This geographic location strategically complies with the principal mission of this hospital, which is to provide healthcare support to economically deprived rural areas of the country.

Since November 2002, when the cardiac centre became operational, a significant number of patients have presented at the hospital and been treated for various pathologies.^4^ Obviously, treatment and prevention of malaria and HIV, and vaccination programmes are top of the list of priorities for public health in Cameroon. However, in our personal experience to date, we know that in Cameroon there are a significant number of patients with diagnosed or undiagnosed congenital heart diseases who are helpless because of associated expensive treatment.

The only scientifically based approach to draw public health attention to the seriousness of the problem is to provide evidence-based data that support our observations. Currently, there are no epidemiological data available on the prevalence and management of congenital heart diseases in Cameroon, and only a limited number of published studies on rural areas of sub-Saharan Africa.^5^,^6^ Therefore, the aim of this study was to determine the occurrence and pattern of congenital heart diseases in the cardiac centre of St Elizabeth Catholic General Hospital, situated in a rural area of Cameroon.

## Methods

The ethics committee of St Elizabeth Catholic General Hospital approved the study. Between November 2002 and November 2008, a population of 2 123 patients aged between two months and 41 years (mean: 10.03 ± 9.7 years) was seen in the cardiac centre for various pathologies. Patients with one or a combination of the following pathological features: precordial murmurs, past history of recurrent respiratory heart diseases, clinical indications of suspicious cardiopathy and/or cardiomegaly on chest X-ray examination (cardiothoracic index > 0.55) underwent further screening tests for detection of congenital heart diseases. A total of 292 patients (58.2% females and 41.8% males) were recruited for the study.

Initially, patients underwent clinical examination, followed by a comprehensive transthoracic Doppler echocardiogram using an Acuson 4–7 MHz. In addition, a complete two-dimensional echocardiography examination was performed according to the recommendations of the American Society of Echocardiography.

Depending on the pathology, patients diagnosed with congenital heart diseases were put on a surgical list for sanitary evacuation to a collaborative centre (Instituto Polyclinico San Donato) in Italy where corrections of pathology were performed. The Tertiary Sisters of St Francis and two other charity organisations, the Cuore Fratello and Associazione Bambini Cardiopatici nel Mondo, paid for treatments and all inccured costs. Upon returning to Cameroon, patients were followed up at the Shisong cardiac centre.

## Statistical analysis

Values are expressed as mean ± standard deviation (SD) and statistical analyses were performed using the Student’s *t*-test. SPSS 11 statistical analysis software was used for all analyses and a *p*-value ≤ 0.05 was considered significant.

## Results

Two groups of patients were identified based on their diagnoses. The first group (95.5%) had an echocardiographic diagnosis of congenital heart disease (*n* = 279) and the second (4.5%) inorganic murmurs (*n* = 13).

**Fig. 1.**
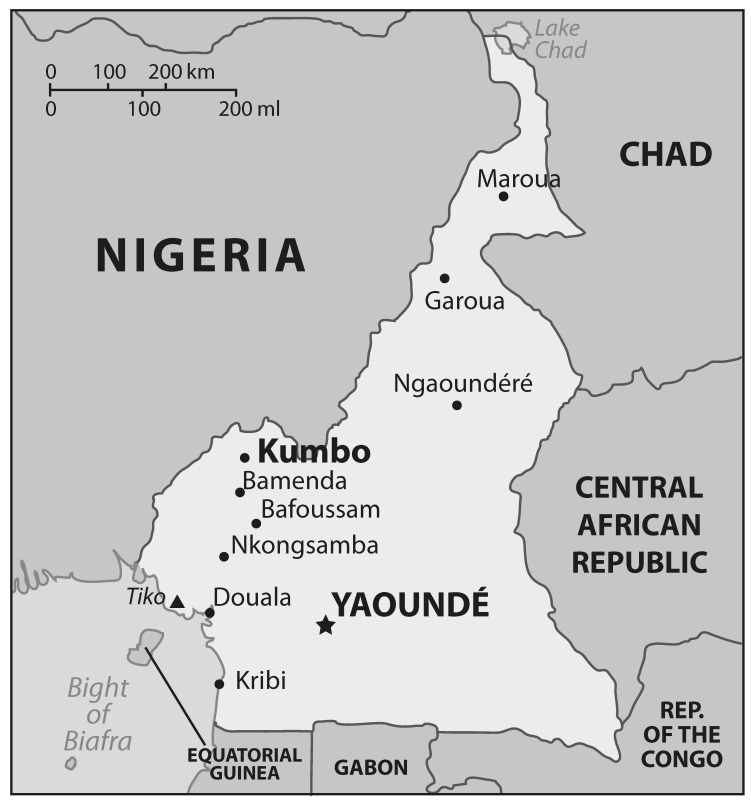
Map of Cameroon showing the provincial areas as well as the neighbouring countries of the patients included in the study.

There are 10 provincial regions in Cameroon and patients’ geographic origins were distributed as follows (Fig. 1): north (Maroua and Garoua, *n* = 2), north-west (Bamenda, *n* = 80), littoral (Douala, Nkongsamba and Tiko, *n* = 89), central (Yaounde, *n* = 66), and west (Bafoussam, *n* = 28) regions. The remaining patients (*n* = 11) were from the other provinces and neighbouring countries such as Gabon (*n* = 1) and Nigeria (*n* = 3). In addition, there are also more than 260 tribes in Cameroon. The patients’ tribal origins were (Table 1): Banso (35.3%), Bamileke (32.2%), Douala (12.7%), Haoussa (8.4%), Ewondo (5.4%), Bakweri (2.6%) and others (3.4%).

**Table 1.** Patient Distribution By Tribe, And Tribal Representation In Cameroon

*Tribe*	*Patients (%)*	*Tribal representation in Cameroon (%)*
Banso	35.3	12.8
Bamileke	32.2	18.3
Douala	12.7	13
Haoussa	8.4	20.2
Ewondo	5.4	17.6
Bakweri	2.6	8
Others	3.4	10.1

Of the 1 831 patients remaining out of the total patients consulting (2 123), 321 had rheumatic heart disease (RHD) while the rest had no heart-related pathologies.

## Transthoracic Doppler echocardiogram

Congenital heart diseases were distributed as follows (Tables 2, 3): tetralogy of Fallot (26.1%), isolated ventricular septal defect (38.8%), atrioventricular cushion defect (7.3%), isolated atrial septal defect (2.8%), arterial duct cases (12.4%), common arterial trunk (1.3%), isolated stenosis of the pulmonary artery (2.6%), coarctation of the aorta (1.1%), congenital mitral valve regurgitation (1.2%), atresia of the triscupid valve (1.6%), double-outlet right ventricle (2.1%), anomalous pulmonary venous return (1.5%) and left isomerism (1.2%).

**Table 2.** Percentage Distribution Of Congenital Heart Diseases Per Pathology (n = 279 Patients)

*Pathology*	*Percentage (%)*
Isolated ventricular septal defect	38.8
Tetralogy of Fallot	26.1
Arterial duct	12.4
Atrioventricular cushion defect	7.3
Isolated atrial septal defect	2.8
Isolated stenosis of the pulmonary artery	2.6
Double-outlet right ventricle	2.1
Atresia of the triscupid valve	1.6
Anormalous pulmonary venous return	1.5
Common arterial trunk	1.3
Congenital mitral valve regurgitation	1.2
Left isomerism	1.2
Coarctation of the aorta	1.1

**Table 3.** Pathology Distribution With Regard To Patient Age At The Time Of Diagnosis

*Pathology*	*Age*		
	*2 months – 2 years (%)*	*2–5 years (%)*	*> 5 years (%)*
Isolated ventricular septal defect	22	14.8	2
Tetralogy of Fallot	10	13.1	3
Arterial duct	6	3.9	2.5
Atrioventricular cushion defect	5	2.3	-
Isolated atrial septal defect	0.9	1.1	0.8
Isolated stenosis of the pulmonary artery	0.9	1.2	0.5
Double-outlet right ventricle	1.8	0.3	-
Atresia of the triscupid valve	1.1	0.5	-
Anormalous pulmonary venous return	0.6	0.7	0.2
Common arterial trunk	0.8	0.5	-
Congenital mitral valve regurgitation	0.2	0.3	0.7
Left isomerism	0.4	0.8	-
Coarctation of the aorta	0.2	0.4	0.5

There was a significantly (*p* ≤ 0.005) high percentage of large isolated peri-membranous ventricular septal defects (85%) compared to restrictive (10.2%) and muscular (4.8%) defects. Complete atrioventricular cushion defects (63.5%) were also significantly higher (*p* ≤ 0.005) than incomplete (39.5%) cushion defects. Combined pathologies, such as ventricular septal defect with isolated atrial septal defect were found in 1.8% (*n* = 5) of patients; ventricular septal defect with arterial duct in 2.1% (*n* = 6); ventricular septal defect with pulmonary valve stenosis in 8.9% (*n* = 25); and left isomerism with arterial duct in 1% (*n* = 3) of cases.

## Pre- and post-surgical follow up

One hundred and eleven patients underwent open-heart surgery in Italy at the Polyclinico San Donato, while 12 patients were operated on at St Elizabeth Catholic General Hospital during organised cardio-surgical missions. One hundred and fifty-eight patients were left on the surgical waiting list for the next surgeries. Four per cent of the patients were lost during follow up and three post-surgical patients died suddenly. In addition, 25 patients died while still on the waiting list.

## Discussion

This study shows that over a period of six years, 2 123 patients were seen at the St Elizabeth Catholic General Hospital cardiac centre and that congenital heart diseases were the most prevalent diagnosed pathologies, found in 13.1% (*n* = 279) of the patients. This observation is in line with previously published studies.

Transthoracic echocardiography data from the retrospective study of Mahmoud *et al*. conducted in two of Kano’s laboratories in Nigeria over a period of 48 months showed that 9.3% (*n* = 122) of the patients (aged 9 days to 35 years old) presenting abnormal echocardiograms had congenital heart diseases.^7^ However, another study by Bassili *et al*. conducted in Egypt showed a low incidence (1.01/1 000) of congenital heart diseases among school children.^8^

Because of its state-of-the-art facility, the Shisong Cardiac Centre, which is relatively new, is a renowned referral centre in the region. Consequently, many patients have been referred by practitioners in various disciplines, including cardiologists, paediatricians and general practitioners from different parts of the country, for better management of heart murmurs, confirmation of suspected diagnosis of congenital heart diseases and/or enrolment in the sanitary evacuation programme. This could explain the difference between our results and those of the Egyptian study.

We found that the most encountered congenital pathologies were isolated ventricular septal defect and tetralogy of Fallot. Both pathologies were also most frequently found by Bannerman and el Haq among patients with congenital heart diseases.^9^,^10^ In addition, Ejim and colleagues showed that isolated ventricular septal defect was the most prevalent pathological condition, being diagnosed in 70% of all the cases of congenital heart diseases.^11^ Acyanotic congenital heart diseases were more prevalent than cyanotic heart diseases. Cushion defects were mostly diagnosed in patients with trisomy 21.

The majority of these patients could not afford treatment due to the high cost and, as evidence of the difficulty in accessing healthcare, numerous late presentations to practitioners were registered. In a previous study by Ariane *et al*., late presentation to practitioners was observed in 79.3% of cases.^12^ Both access to healthcare and the high cost of treatment also explain the very high incidence of late presentations (68.2%) recorded in Bannerman’s study in Zimbabwe.^9^

We diagnosed a peri-membranous ventricular septal defect and a tetralogy of Fallot in a 13- and 16-year-old boy and girl, respectively. Adults with natural evolutive congenital heart diseases were found in 1.5% of cases.

In the sub-Saharan region of Africa, St Elizabeth Catholic General Hospital has registered the highest percentage (41%, *n* = 114) of cardiac surgeries realised abroad. Previous studies from Senegal,^13^ Sudan,^14^ Mauritania,^15^ and Tunisia^16^ have reported respectively, that only 17.3% (*n* = 75), 28% (*n* = 435), 26% (*n* = 61), and 22.5% (*n* = 12) of the patients who needed a cardiac surgical intervention actually had one done. Updated data from these countries could help clarify the state of the situation to date.

Although the cardiac centre of St Elizabeth Catholic General Hospital is relatively new, it has been the point of attraction for a growing number of new patients from neighbouring countries. Cameroon is a bilingual nation characterised by a French- and English-speaking region. However, despite the cardiac centre being located in the English-speaking region, more than 60% of the consulting patients come from the French-speaking region.

In a country with over 260 tribes, all patients are seen irrespective of their tribe and/or religious beliefs. In that respect, the hospital is a crossroads of several tribes. It might seem taboo, but it is important to highlight the multi-tribal orientation of the hospital as Cameroon is a country where the way someone is treated could be determined by his/her tribal origin, depending if that tribe is well represented or not in society.

We also found that congenital heart diseases were predominant in the Banso and Bamileke people. However, this could be due to the fact that the majority of patients came from these regions, which are closest to the hospital. Bamileke are known to marry close relatives and therefore we could hypothesise that genetic background could be linked to the predominance of congenital heart diseases found in this tribe. However, unless scientifically proven, this must remain an assumption.

Since there are only a few cardiac centres in the country, and more importantly because patients cannot afford the cost of treatment, pre- and post-surgical follow up is very challenging. There is a need to establish programmes to better inform patients about their disease and the importance of proper follow up. More than 60% of children have a very poor academic background and almost the same percentage of rural parents do not have a secondary school education.

Long-term post-surgical death rate is estimated at 0.7% and the main cause of death is thought to be malignant arrhythmias occurring after correction of the congenital heart disease. In developed countries, particularly Europe and America, diagnosis and treatment of cardiac pathologies are readily affordable by most citizens through coverage by insurance plans. In the majority of developing nations, especially countries on the African continent, this is not yet the case.

Thanks to the positive partnership between St Elizabeth Catholic General Hospital, Policlinico San Donato in Milan, Tertiary Sisters of St Francis, Associazione Bambini Cardiopatici nel Mondo and Cuore Fratello, some hope is offered to patients and their families. The initiative of St Elizabeth Catholic General Hospital in supporting early detection, diagnosis, treatment and patient follow up is encouraging; however, public health involvement and better funding are required to cover the expenses so that all patients are afforded the opportunity to receive proper treatment in a timely manner.

## Conclusion

The data showed that a wide range of congenital heart diseases were represented in the cardiac centre of St Elizabeth Catholic General Hospital, Shisong, situated in a sub-Saharan rural area of Africa, and that isolated ventricular septal defect was the most prevalent pathology. However, despite successful cardiac surgery and treatment, patient follow up remained a significant challenge.
